# Phase Equilibria and Magnetic Phases in the Ce-Fe-Co-B System

**DOI:** 10.3390/ma10010016

**Published:** 2016-12-28

**Authors:** Tian Wang, Dmytro Kevorkov, Mamoun Medraj

**Affiliations:** 1 Department of Mechanical and Industrial Engineering, Concordia University, 1455 de Maisonneuve Boulevard West, Montreal, QC H3G 1M8, Canada; wa_ti@encs.concordia.ca (T.W.); dmytro.kevorkov@gmail.com (D.K.); 2 Department of Mechanical and Materials Engineering, Masdar Institute, Masdar City P.O. Box 54224, Abu Dhabi, United Arab Emirates

**Keywords:** phase equilibria, Ce-Fe-Co-B quaternary system, magnetic phases, MFM, SEM/WDS

## Abstract

Ce-Fe-Co-B is a promising system for permanent magnets. A high-throughput screening method combining diffusion couples, key alloys, Scanning Electron Microscope/Wavelength Dispersive X-ray Spectroscope (SEM/WDS), and Magnetic Force Microscope (MFM) is used in this research to understand the phase equilibria and to explore promising magnetic phases in this system. Three magnetic phases were detected and their homogeneity ranges were determined at 900 °C, which were presented by the formulae: Ce_2_Fe_14−*x*_Co*_x_*B (0 ≤ *x* ≤ 4.76), CeCo_4−*x*_Fe*_x_*B (0 ≤ *x* ≤ 3.18), and Ce_3_Co_11−*x*_ Fe*_x_*B_4_ (0 ≤ *x* ≤ 6.66). The phase relations among the magnetic phases in this system have been studied. Ce_2_(Fe, Co)_14_B appears to have stronger magnetization than Ce(Co, Fe)_4_B and Ce_3_(Co, Fe)_11_B_4_ from MFM analysis when comparing the magnetic interactions of selected key alloys. Also, a non-magnetic CeCo_12−*x*_Fe*_x_*B_6_ (0 ≤ *x* ≤ 8.74) phase was detected in this system. A boron-rich solid solution with Ce_13_Fe*_x_*Co*_y_*B_45_ (32 ≤ *x* ≤ 39, 3 ≤ *y* ≤ 10) chemical composition was also observed. However, the crystal structure of this phase could not be found in the literature. Moreover, ternary solid solutions ε_1_ (Ce_2_Fe_17−*x*_Co*_x_* (0 ≤ *x* ≤ 12.35)) and ε_2_ (Ce_2_Co_17−*x*_Fe*_x_* (0 ≤ *x* ≤ 3.57)) were found to form between Ce_2_Fe_17_ and Ce_2_Co_17_ in the Ce-Fe-Co ternary system at 900 °C.

## 1. Introduction

Manufacturing of hybrid and electric cars has escalated the need for strong and inexpensive permanent magnets. Modern permanent magnets devices require the presence of large coercivity. Among different commercial permanent magnets, rare-earth-based materials have been considered as the most favorable candidates due to their suitable magnetic properties [1]. The growing world demand for permanent magnets and rising costs of Nd and other less abundant rare earth metals (e.g., Sm and Dy) necessitates the development of new Fe-based magnets with the addition of relatively abundant inexpensive rare earth metals (e.g., Ce). Despite the low Curie temperature, Ce_2_Fe_14_B was found to have suitable magnetic properties and could possibly be used in the industry [2]. Co was recognized as a potential additive to increase Curie temperature of Ce_2_Fe_14_B [3]. Thus, the Ce-Fe-Co-B quaternary system is a promising system for magnetic materials with suitable magnetic properties as well as low cost for the automotive or electromechanical applications, especially in the Fe-rich region. Knowledge of phase equilibria is necessary for magnetic materials development and for the optimization of magnets composition as well as establishing the heat-treatment conditions. However, limited experimental results can be found in the literature regarding this system. In this research, phase equilibria in the Ce-Fe-Co-B system are investigated. For this purpose and to find promising magnetic compounds, a high-throughput screening (HTS) method is adopted.

## 2. Literature Review

### 2.1. Ternary Systems

The Ce-Fe-Co-B system consists of Ce-Fe-B, Co-Fe-B, Ce-Co-B, and Ce-Fe-Co four sub-ternary systems. The phase equilibria in Ce-Fe-B, Co-Fe-B, and Ce-Co-B were established or partially established in the literature. A number of ternary compounds have been found among these three systems. However, there are still some questionable and uncertain phase relationships in the Ce-Fe-Co system which will be described in the coming section. A brief literature review regarding all the constituent ternary systems is included below.

**Figure 1:**
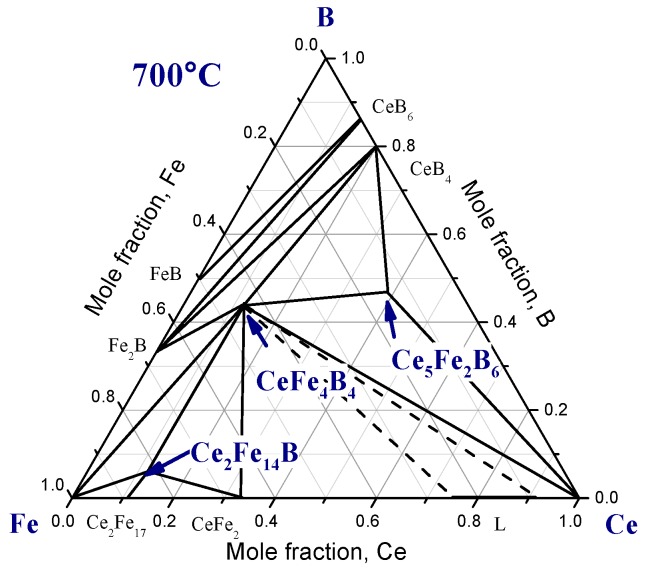
Isothermal section of Ce-Fe-B system at 700 °C [8].

The phase diagram of the Ce-Fe-B system was investigated by several researchers [4,5,6,7]. Raghavan [8] summarized the previous works and re-drew the isothermal section at 700 °C, as shown in Figure 1. As can be seen from this figure, three ternary compounds, Ce_2_Fe_14_B, CeFe_4_B_4_, and Ce_5_Fe_2_B_6_, were reported.

**Figure 2:**
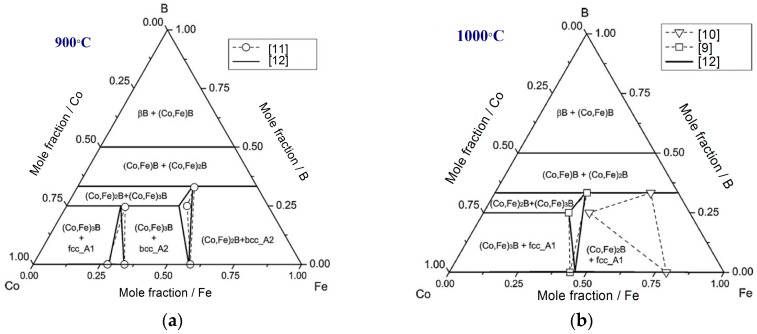
Isothermal section of Co-Fe-B ternary system at (**a**) 900 °C [12]; and (**b**) 1000 °C [12].

The early experimental studies of the Co-Fe-B ternary system can be found in [9,10,11]. Liu et al. [12] calculated isothermal sections of this system at 900 °C and 1000 °C. Their results were in reasonable agreement with the experimental data provided by Van Loo et al. [11] at 900 °C as shown in Figure 2a. At 1000 °C, the experimental data provided by Liu et al. [12] and Rogl et al. [9] contradicted those reported by of Pradelli et al. [10], as can be seen from Figure 2b.

**Figure 3:**
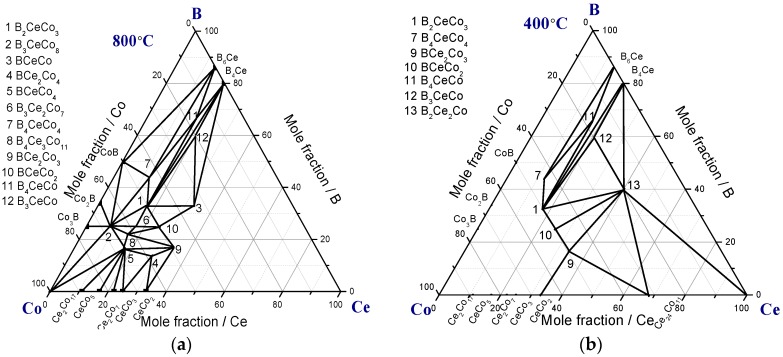
Isothermal section of B-Ce-Co ternary system at (**a**) 800 °C and (**b**) 400 °C redrawn from [13].

Two partial isothermal sections of Ce-Co-B were reported by Bilonizhko et al. [13], as shown in Figure 3. Thirteen ternary compounds (B_2_CeCo_3_, B_3_CeCo_8_, BCeCo, BCe_2_Co_4_, BCeCo_4_, B_3_Ce_2_Co_7_, B_4_CeCo_4_, B_4_Ce_3_Co_11_, BCe_2_Co_3_, BCeCo_2_, B_4_CeCo, B_3_CeCo, and B_2_Ce_2_Co) were found. It was reported that BCeCo is a high-temperature compound that formed at 800 °C and decomposed at 400 °C. Therefore, BCeCo can be seen only in Figure 3a, not in Figure 3b. Later, four additional ternary compounds were reported: B_2_Ce_2_Co_5_ [14], BCe_3_Co_20_ [15], B_6_CeCo_12_ [16], and B_6_Ce_5_Co_2_ [17]. However, the thermal stability of these compounds and their phase relationships are still unknown. Having sixteen ternary compounds makes B-Ce-Co a very complex system. Therefore, a significant amount of experimental and thermodynamic modeling research on this system is still needed.

**Figure 4:**
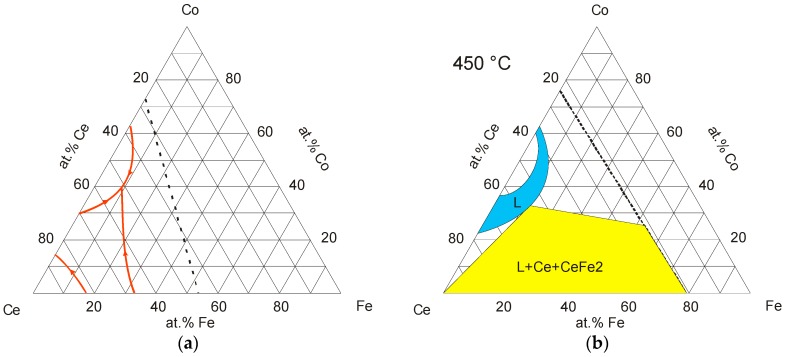
(**a**) Partial liquidus projection of Ce-Co-Fe system [18]; (**b**) partial isothermal section of Ce-Co-Fe system at 450 °C [18].

The literature data on the experimental phase equilibria in the Ce-Co-Fe ternary system is limited to the work of Critchley [18], who reported a partial liquidus projection (Figure 4a) and partial triangulation of the isothermal section at 450 °C (Figure 4b). These figures were re-drawn from the ASM Alloy Phase Diagram Database [19]. It should be mentioned that the triangulation of the L + Ce + CeFe_2_ three-phase region in Figure 4b is incorrect, because CeFe_2_ compound must not extend beyond 33 at. % Ce at the Ce-Fe side, according to the chemical composition of the compound. Also, the liquid region in the Ce-Co system should be limited to the range of 22–34 at. % Co, based on the most recent Ce-Co binary phase diagram [20]. Moreover, Mansey et al. [21] studied the change of lattice parameters of the CeFe_2_-CeCo_2_ quasi-binary section. They reported a continuous solid solubility between the CeFe_2_ and CeCo_2_ compounds. These results were confirmed later by Harris and Longworth [22], and Longworth and Harris [23], who reported that the CeFe_2_-CeCo_2_ solid solution at 1173 K has a cubic C15 Laves phase. In this ternary system, the phase equilibria in the Co-Fe side is still unclear. Especially, the phase relationships between Ce_2_Co_17_ and Ce_2_Fe_17_ are missing in the literature. These two compounds are isostructural compounds with a hexagonal Th_2_Zn_17_ crystal structure prototype [24]. Therefore, they are expected to form a continuous solid solution.

### 2.2. Quaternary System

Limited experimental results regarding the phase equilibria in the Ce-Fe-Co-B system can be found in the literature. Skoug et al. [3] reported two series of Ce_3_Fe_14−*x*_Co*_x_*B and Ce_2.55_Fe_14−*x*_Co*_x_*B_1.27_ melt-spun ribbons and their magnetic properties were also studied. Co was found to dissolve in Ce_2_Fe_14_B and form magnetic Ce_2_(Fe, Co)_14_B compound. However, the maximum solid solubility of Ce_2_(Fe, Co)_14_B is still unknown, and the phase relationships need to be understood. Also, the presence of other magnetic phases in this system should be verified. Thus, it is necessary to carry out research to understand the phase equilibria as well as to screen other promising magnetic phases in the Ce-Fe-Co-B system.

## 3. Materials and Methods

By combining diffusion couples and key alloys, with the aid of the Scanning Electron Microscope (SEM) and Magnetic Force Microscope (MFM), the HTS method is much more efficient to understand the phase equilibria and to screen magnetic compounds in a multi-component system. HTS is used to screen magnetic phases in the Ce-Fe-Co-B multi-component system combining microstructural, micro-elemental, and magnetic domain analyses of diffusion couples and key alloys. Magnetic phases could be identified through their domain interactions with the magnetic tip of the MFM. The analysis of one successful diffusion couple can potentially give complete information on a large number of intermetallic phases in the system at a specific temperature. Based on the diffusion couples results, key alloys are prepared and studied further by X-ray Diffraction (XRD) for phase identification and MFM to confirm the presence of a magnetic phase in a significant amount. Importantly, the key alloys can be used to measure intrinsic magnetic properties, such as saturation magnetization, anisotropy fields, and Curie temperature. These properties for Ce_2_Fe_14_B modified by Co will be reported in another paper. HTS significantly reduces the number of experiments and the timeframe. However, ternary or quaternary diffusion couples have unpredictable diffusion paths, which could lead to omitting some phases. Besides, slow kinetic formation of some phases may cause the formation of thin layers that might be difficult to be successfully analyzed [25]. Therefore, key alloys are used to verify the results obtained from diffusion couples. In this study, four solid-state diffusion couples and fifteen key alloys were prepared. Pure materials with 99 wt. % purity or better are used as the starting materials. All the elements are supplied by Alfa Aesar^®^ (Haverhill, MA, USA), Johnson Matthey Company (London, UK). Samples of known composition are prepared from pure metals using an arc melting furnace under argon atmosphere.

The arc melting furnace is equipped with a water-cooled copper crucible and a non-consumable tungsten electrode. Every alloy must be melted several times to ensure homogeneity. The prepared samples were used as key alloys or as end-members for the diffusion-couple experiments. Diffusion couples were prepared by grinding down the contacting interfaces of end-members using 1200 grit SiC paper and then polished up to 1 µm using alcohol-based diamond suspension. Ninety-nine per cent pure ethanol was used as lubricant. The selected end-members were carefully pressed and clamped together using a stainless steel ring. For the annealing process, samples were encapsulated inside quartz tubes under vacuum. After sufficient annealing time (at least 25 days), samples were quenched in a cold water bath to obtain the high-temperature structure. Quenched samples or diffusion couples were polished and grinded up to 1 µm in order to be analyzed using SEM coupled with Energy/Wavelength Dispersive X-ray Spectroscopes (EDS/WDS) and MFM. The microstructure and phase composition of the samples were analyzed by SEM/WDS (HITACHI S-3400N, HITACHI, Tokyo, Japan). XRD was performed for the key alloys using PANAnalytical Xpert Pro X-ray diffractometer (PANAnalytical, Almelo, The Netherlands) with a CuKα radiation at 45 kV and 40 mA. XRD patterns were analyzed using X’Pert Highscore plus software [26] and the Rietveld method. The crystal structure prototypes of the detected phases were obtained from Pearson’s Database [24] and used in XRD analysis. Quenched samples were also used for MFM imaging using Digital Instruments Multimode Atomic Force Microscope (Digital Instruments, Billerica, MA, USA) in LiftMode. In this work, a 225 µm long silicon cantilever having a magnetic pyramidal tip was used to acquire the magnetic gradient images. The MFM tip, supplied by Appnano Ltd. (Mountain View, CA, USA) was coated with an approximately 50 nm CoCr layer. The cantilevers have resonant frequency between 47 and 76 kHz. This frequency is shifted by an amount proportional to the vertical gradient of the magnetic forces on the tip [27]. The frequency shifts can be detected by the phase detection capability of the MFM. The drive frequency of the cantilever for the phase detection was set to be the center of the cantilever resonance. The signal was measured as the cantilever’s phase of oscillation relative to the piezo drive. And the magnetic contrast can be achieved through the magnetostatic interaction between the MFM tip and the stray fields from the sample [27]. When attractive interactions occur, negative phase shift and dark image contrast can be observed in the MFM images. Whereas, positive phase shift and bright image contrast result when repulsive interactions take place [27].

## 4. Results and Discussion

### 4.1. Diffusion Couples Results

In order to understand the phase equilibria in the Ce-Fe-Co-B system, four diffusion couples were prepared. The chemical compositions across the diffusion couples and the corresponding phases that formed in the diffusion layers are listed in Table 1. Diffusion couples were all annealed at 900 °C for 4 weeks.

**Figure 5:**
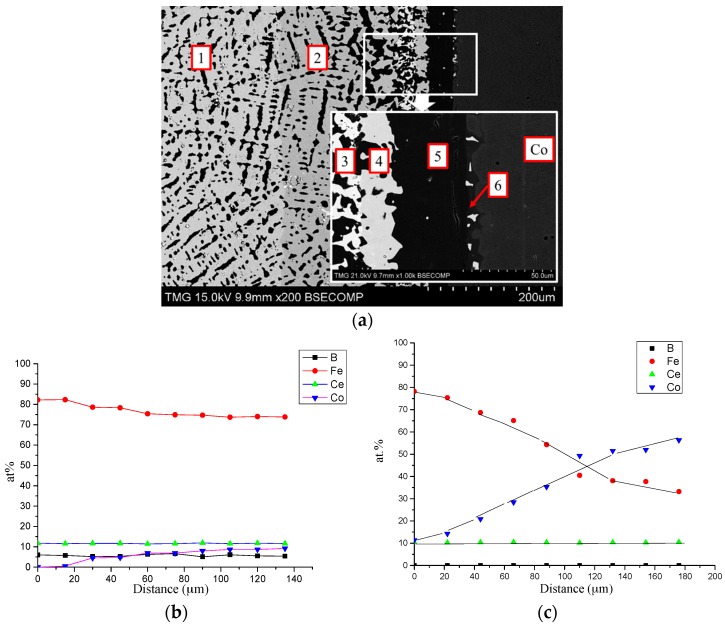
(**a**) BSE image of DC1; (**b**) WDS compositional profile of Ce_2_(Fe, Co)_14_B in layer 1; (**c**) WDS compositional profile of Ce_2_(Fe, Co)_17_ in layer 2.

The backscattered electron (BSE) image of DC1 is presented in Figure 5a. As a result of diffusion between Ce_2_Fe_14_B and Co, six diffusion layers formed. The compositions of the formed phases were determined using WDS point analysis. The solubility ranges were measured by WDS line scan. In the first layer, the end-member Ce_2_Fe_14_B (white) was in equilibrium with the α-(Fe, Co) phase (black). Fe was substituted by Co in both phases during the diffusion process. A WDS compositional profile of Ce_2_(Fe, Co)_14_B is shown in Figure 5b. The quaternary solid solubility of Ce_2_Fe_14_B was determined as 9 at. % Co, which was presented by the formula Ce_2_Fe_14−*x*_Co*_x_*B (0 ≤ *x* ≤ 1.54). Co in α-Fe in layer 1 was found to be 6 at. %. The grey phase in layer 2 was identified as Ce_2_(Fe, Co)_17_ with Ce_11_Fe_34–79_Co_10–55_ composition and named as ε_1_ in this study. The WDS compositional profile of Ce_2_(Fe, Co)_17_ is plotted in Figure 5c. At the boundary between layers 1 and 2, three phase equilibrium (Ce_2_(Fe, Co)_14_B/α-(Fe, Co)/ε_1_) was observed. Moreover, the concentration of Co dissolved in α-Fe phase (black) also gradually increased from 6 to 44 at. % in layer 2. White phase in layer 3 was found to be a stoichiometric compound with Ce_16_Fe_9_Co_59_B_16_ composition. This new quaternary compound is in equilibrium with α-(Fe, Co) (black) and had similar composition to CeCo_4_B, considering the Fe-Co substitution which was also confirmed by key alloys. A three-phase region was established between ε_1_, α-(Fe, Co), and Ce(Co, Fe)_4_B. Co-rich Ce_2_(Co, Fe)_17_ with Ce_11_Fe_19_Co_70_ chemical composition was detected in layer 4, where it is in equilibrium with α-(Fe, Co). Substitution of Fe by Co was found to be up to 61 at. %. Another three-phase region was obtained among α-(Fe, Co), Ce(Co, Fe)_4_B, and Ce_2_(Co, Fe)_17_. Through WDS analysis, layer 5 has been analyzed as α-(Fe, Co) and its homogeneity range was measured as 65–70 at. % Co. The dominating black phase in the thin layer 6 was determined to be γ-(Fe, Co) with a relatively small amount of Fe of about 8–12 at. %. This phase is in equilibrium with Ce_2_(Co, Fe)_17_ (white), which was measured to have Ce_11_Fe_2_Co_87_ chemical composition. This two-phase equilibrium is also confirmed by DC2.

**Figure 6:**
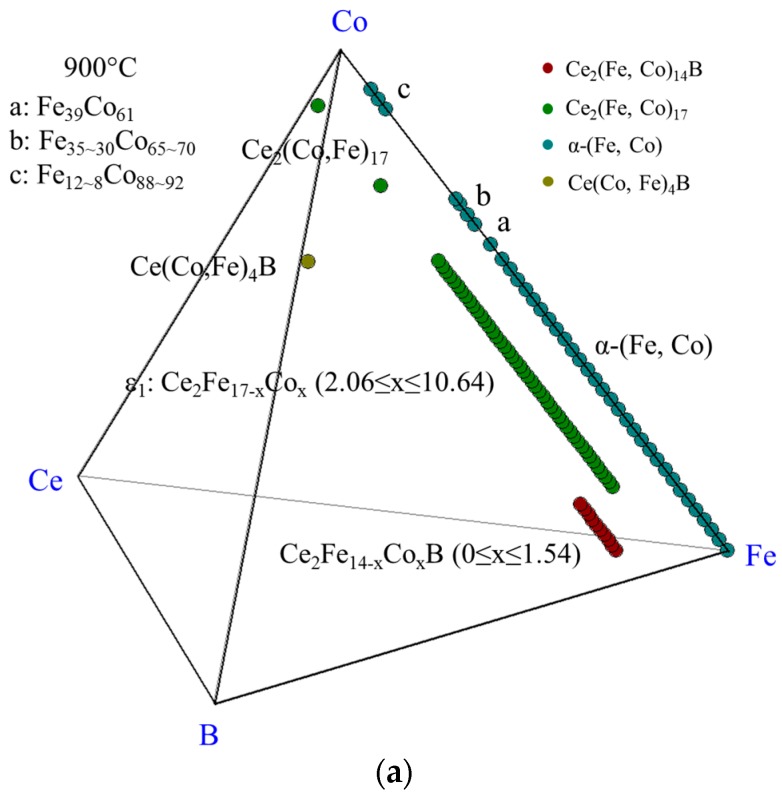

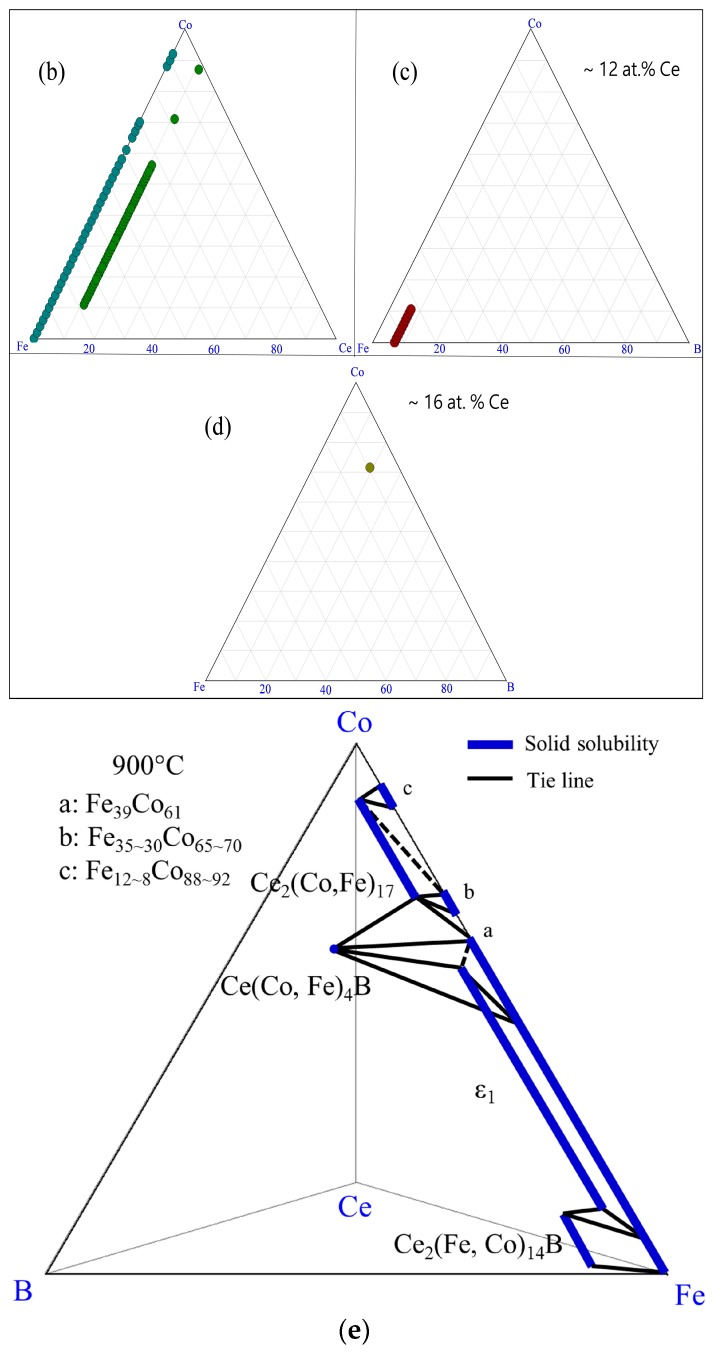
The results of the diffusion couple 1 at 900 °C: (**a**) 3D presentation of the experimental results; (**b**) Fe-Ce-Co ternary system; (**c**) pseudo ternary section at ~12 at. % Ce; (**d**) pseudo ternary section at ~16 at. % Ce; (**e**) phase relations obtained from DC1.

The experimental results of DC1 are summarized in the 3D view, as shown in Figure 6a. The phases which were detected in this diffusion couple are denoted by different colors. The ε_1_ and α-(Fe, Co) are illustrated on the Fe-Co-Ce ternary system in Figure 6b. Two pseudo ternary sections at 12 at. % and 16 at. % Ce are plotted in Figure 6c,d to enable better understanding of the phase relations in DC1. The phase relations obtained from DC1 are summarized in Figure 6e. Three three-phase regions, Ce_2_(Fe, Co)_14_B/α-(Fe, Co)/ε_1_, α-(Fe, Co)/ε_1_/Ce(Co, Fe)_4_B, and Ce(Co, Fe)_4_B/α-(Fe, Co)/CeFe_2_Co_7_, are established at the interfaces from layers 1–4 of DC1.

Ce_13_Fe_80_B_7_ and Co_90_Ce_10_ were selected as the end-members of DC2. By doing so, the diffusion path may cross several phase regions, and Fe/Co atomic exchange in Ce_2_(Fe, Co)_14_B can be further understood. The Ce_2_(Fe, Co)_14_B phase was confirmed in DC2 and solid solubility was measured as 22 at. % Co which was greater than DC1. Two layers of Ce(Co, Fe)_4_B were observed in DC2, with solid solubility of 17–27 at. % Fe and 1–6 at. % Fe. From SEM/WDS analysis, as listed in Table 1, a single α-(Fe, Co) layer (layer 3) formed in between of two Ce(Co, Fe)_4_B layers (layers 2 and 4) which could hinder the Co/Fe atomic exchange in Ce(Co, Fe)_4_B. This is why two layers of this solid solution were obtained instead of a continuous Ce(Co, Fe)_4_B layer. Ce_2_(Co, Fe)_17_ and γ-(Fe, Co) two-phase equilibrium was observed and confirmed in layer 5 of DC2, as presented in Table 1. By analyzing DC3, Ce_3_(Co, Fe)_11_B_4_ and Ce(Co, Fe)_12_B_6_ were found to form in this quaternary system at 900 °C and their solid solubilities were measured as 5–21 at. % Fe and 11 at. % Fe, respectively, as can be seen in Table 1. Later, these two phases were all confirmed by DC4.

**Figure 7:**
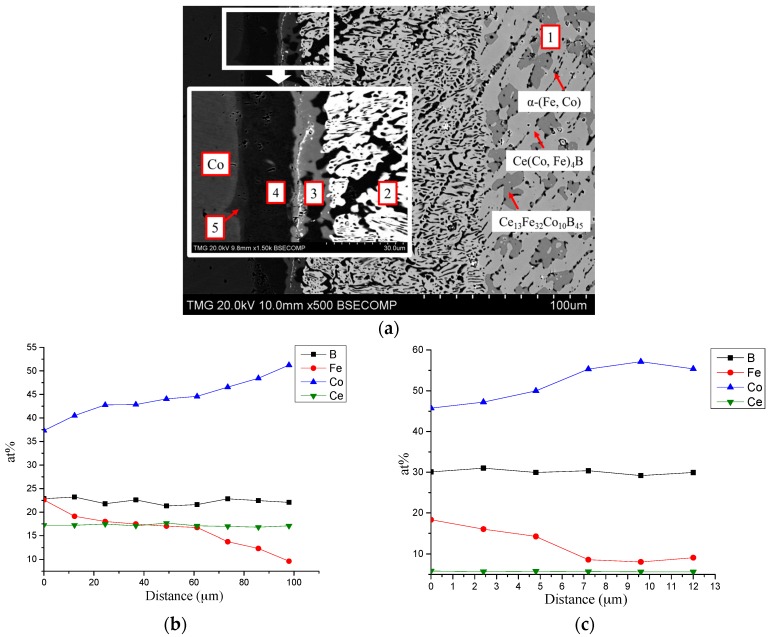
(**a**) BSE image of DC4; (**b**) WDS compositional profile of Ce_3_(Co, Fe)_11_B_4_ in layer 2; (**c**) WDS compositional profile of Ce(Co, Fe)_12_B_6_ in layer 3.

DC4 was designed by combining relatively high Co content Ce_15_Fe_43_Co_19_B_23_ alloy with a pure Co piece. It is impossible to control the diffusion path of diffusion couples. Therefore, it is very essential to select the proper end-members of diffusion couples in order to cross a large number of phase regions, so that more phase equilibria results could be revealed. The BSE image of DC4 is presented in Figure 7a, the Ce_15_Fe_43_Co_19_B_23_ end-member was found to be in the Ce_13_Fe_32_Co_10_B_45_/Ce(Co, Fe)_4_B/α-(Fe, Co) three-phase region. The homogeneity range of Ce(Co, Fe)_4_B was determined as 40–45 at. % Fe. A new boron-rich compound, Ce_13_Fe_32_Co_10_B_45_, was found and more studies have been performed by key alloys to further analyze this compound which will be discussed in the next section. As shown in the enlarged inset of Figure 7a, four diffusion layers are present. From WDS analysis, the composition of the white phase in layer 2 was measured as Ce_17_Fe_23–9_Co_37–51_B_23_, as presented in the WDS compositional profile in Figure 7b. Similar to DC3, this phase was identified as Ce_3_(Co, Fe)_11_B_4_, which was also confirmed by key alloys. Layer 3 mainly contained a Ce(Co, Fe)_12_B_6_ phase which had Ce_6_Fe_18–9_Co_46–55_B_30_ composition. The results of WDS compositional analysis of layer 3 are plotted in Figure 7c. From DC3 and DC4, a Ce(Co, Fe)_12_B_6_ phase is confirmed. Key alloys are used to measure its maximum solubility at 900 °C. A thick layer number 4, which contained α-(Fe, Co) with Fe_31_Co_69_ composition, formed in this diffusion couple.

**Figure 8:**
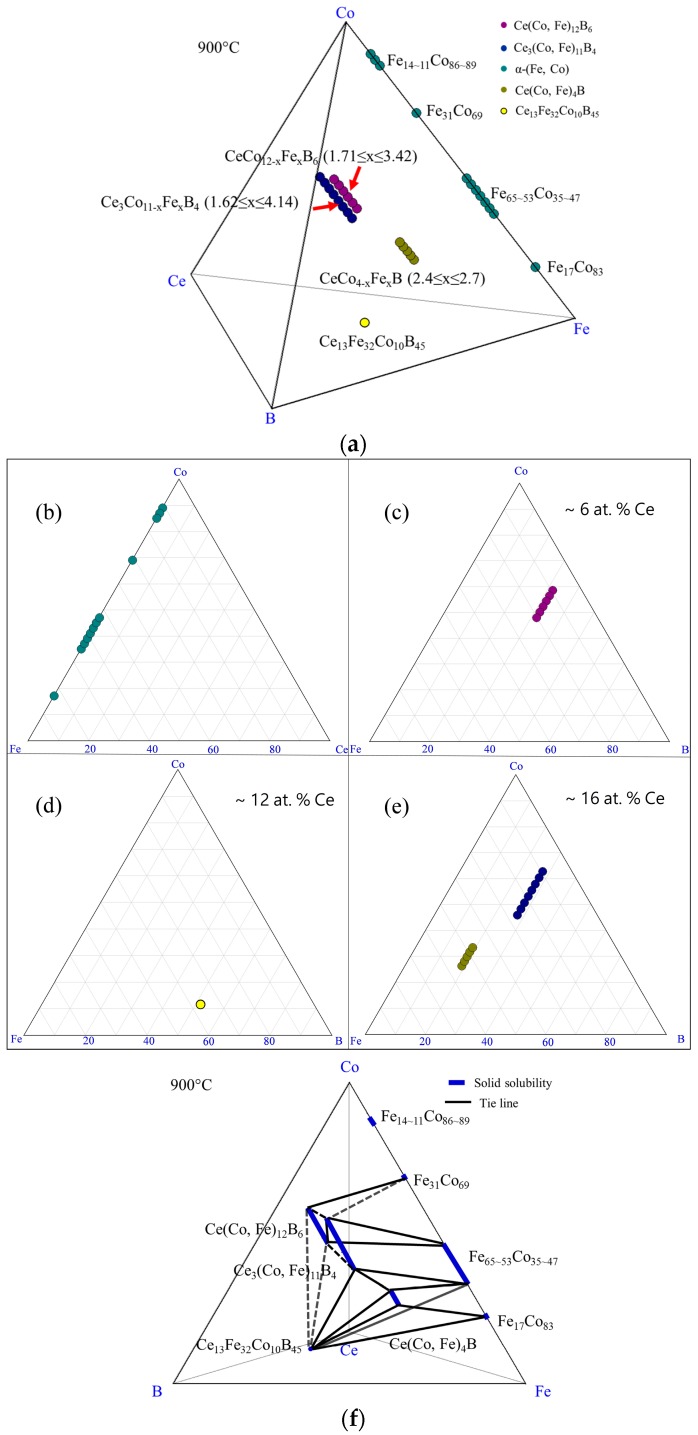
The results of the diffusion couple 4 at 900 °C: (**a**) 3D presentation of the experimental results; (**b**) Fe-Ce-Co ternary system; (**c**) pseudo ternary section at ~6 at. % Ce; (**d**) pseudo ternary section at ~12 at. % Ce; (**e**) pseudo ternary section at ~16 at. % Ce; (**f**) phase relations obtained from DC4.

The experimental results of DC4 are summarized in the 3D view shown in Figure 8a. Different phases are demonstrated by different colors. The α-(Fe, Co) phase is projected on a Fe-Co-Ce ternary system in Figure 8b. The phases detected from this diffusion couple are illustrated by three pseudo ternary sections at 6 at. %, 12 at. %, and 16 at. % Ce, which are shown in Figure 8c–e. The phase relations in DC4 are plotted in Figure 8f. Solid lines represent the phase regions established from DC4 and dotted lines indicate possible phase equilibria among detected phases.

**Figure 9:**
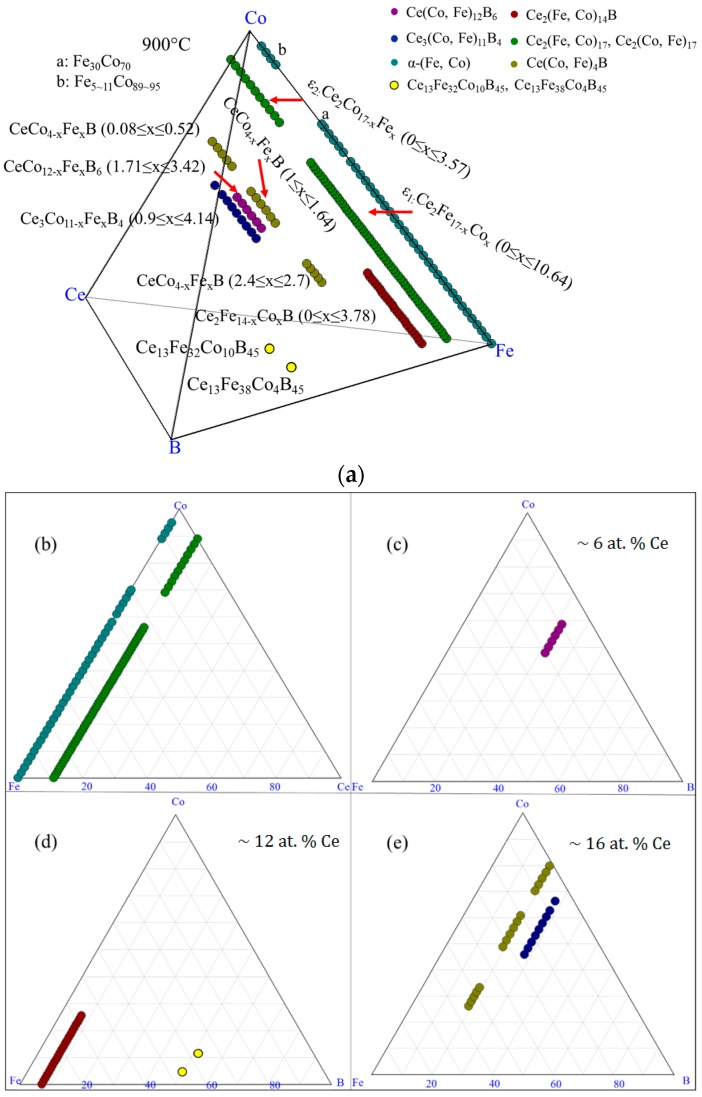
The diffusion couples results of the Ce-Fe-Co-B system at 900 °C: (**a**) 3D presentation of the experimental results; (**b**) Fe-Ce-Co ternary system; (**c**) pseudo ternary section at ~6 at. % Ce; (**d**) pseudo ternary section at ~12 at. % Ce; (**e**) pseudo ternary section at ~16 at. % Ce.

By combining the results of all the diffusion couples, the detected phases in the Ce-Fe-Co-B system at 900 °C are plotted in Figure 9a. The phases present in the Fe-Co-Ce system are presented in Figure 9b. Three pseudo ternary sections at around 6 at. %, 12 at. %, and 16 at. % Ce are also shown in Figure 9c–e, respectively. So far, Ce_2_(Fe, Co)_14_B was found to have solid solubility of 22 at. % Co and presented as Ce_2_Fe_14−*x*_Co*_x_*B (0 ≤ *x* ≤ 3.78). Solid solubility of Ce(Co, Fe)_4_B was measured from the diffusion couples. Further analysis will be performed using key alloys to measure its solubility limit and will be discussed below. Ce(Co, Fe)_12_B_6_ and Ce_3_(Co, Fe)_11_B_4_ were found to form in the Ce-Fe-Co-B system at 900 °C. They are denoted as CeCo_12−*x*_Fe*_x_*B_6_ (1.7 ≤ *x* ≤ 3.42) and Ce_3_Co_11−*x*_Fe*_x_*B_4_ (0.9 ≤ *x* ≤ 4.14), respectively. Two solid solutions, namely, ε_1_ and ε_2_, with the same crystal structure, were found to form between Ce_2_Co_17_ and Ce_2_Fe_17_ in the Ce-Fe-Co ternary system at 900 °C, as shown in Figure 9a,b. Fujii et al. [28] studied the Ce-Fe-Co system and reported the Ce_2_Co_13_Fe_4_ compound. Although this compound is not observed in the current work, its composition is very close to the solubility limit of ε_2_ that has the Ce_2_Co_13.4_Fe_3.6_ formula. Therefore, it could be that the compound reported in the work of Fujii et al. [28] refers to the solubility limit of ε_2_.

### 4.2. MFM Study on Diffusion Couples

**Figure 10:**
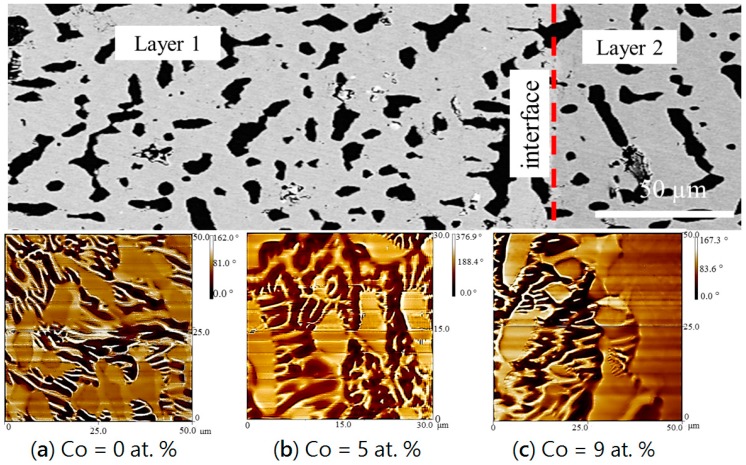
Microstructure (**top**) and three MFM images (**a**–**c**) obtained from layer 1 to the interface with layer 2 of DC1.

The strength of the near-surface stray fields is sensitive to the crystal orientation [29]. Since the investigated samples are non-oriented, various types of domain patterns are present. Diffusion layers of diffusion couple 1 were investigated using MFM. A stripe domain pattern of Ce_2_Fe_14−*x*_Co*_x_*B (0 ≤ *x* ≤ 1.54) in layer 1 was obtained and shown in Figure 10. Three tests were performed starting from the edge of layer 1 to the interface between layers 1 and 2. As presented in Figure 10, three MFM images positively indicate that Ce_2_Fe_14−*x*_Co*_x_*B (0 ≤ *x* ≤ 1.54) is magnetic along its homogeneity range. Figure 10c shows the MFM image taken at the interface of layers 1 and 2. The magnetic domain pattern of Ce_12_Fe_73_Co_9_B_6_ (Ce_2_Fe_14−*x*_Co*_x_*B (*x* = 1.54)) is shown on the left side of Figure 10c; strong magnetic contrast is detected. Comparatively, magnetic interaction of ε_1_ was weak and no strong magnetic contrast can be seen on the right side of Figure 10c.

**Figure 11:**
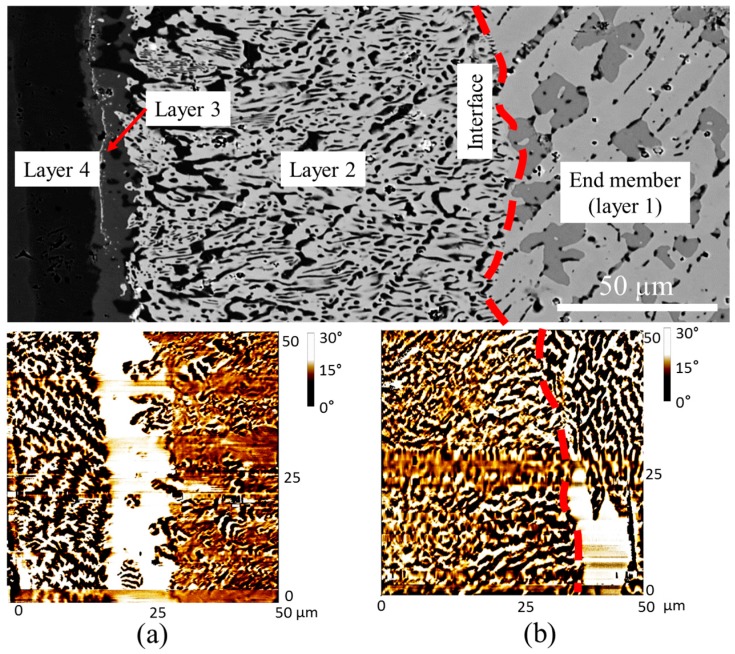
Microstructure (**top**) and two MFM images: (**a**) MFM test on layers 2–4 of DC4; (**b**) MFM test on the interface between layers 1 and 2 of DC4.

An MFM study was also performed on DC4. Two tests were selected to examine the regions of layers 1 and 2, and layers 2–4 as shown in Figure 11. From the MFM results illustrated in Figure 11a, magnetic contrasts of layers 2–4 were captured. On the left side, α-(Fe, Co) with the Fe_31_Co_69_ composition is found to be magnetic and the stripe domain pattern is clearly observed. Also, Ce(Co, Fe)_12_B_6_ in layer 3 is non-magnetic, because no magnetic contrast can be seen in the MFM analysis. Figure 11b is the MFM image at the interface between the end-member (Ce_15_Fe_43_Co_19_B_23_) and layer 2; stronger magnetic contrast was observed at the end-member side (Ce(Co, Fe)_4_B), which has a mixture of stripe and closure magnetic domain patterns. Comparatively, a similar domain pattern is observed in layer 2 (Ce_3_(Co, Fe)_11_B_4_), but the magnetic interaction is weaker. The boron-rich phase, Ce_13_Fe_32_Co_10_B_45_, in the end-member is non-magnetic. Complete analysis of the effect of Co solubility on the intrinsic magnetic properties of the observed phases will soon be reported elsewhere.

### 4.3. Key Alloys Study

#### 4.3.1. Homogeneity ranges of Ce_2_(Fe, Co)_14_B and Ce(Co, Fe)_4_B

From diffusion couple studies, Ce_2_(Fe, Co)_14_B and Ce(Co, Fe)_4_B exhibited extended homogeneity ranges in the Ce-Fe-Co-B system at 900 °C. Eight key alloys were prepared along the homogeneity range of Ce_2_(Fe, Co)_14_B by substituting a different amount of Fe with Co, as listed in Table 2. The actual global compositions of the samples were determined by EDS area mapping. Three maps were taken for each sample. Also, the differences in the three scans were less than 2 at. % for all the elements. All key alloys were annealed at 900 °C for 25 days. Phase equilibria obtained from the key alloys are also presented in Table 2.

**Figure 12:**
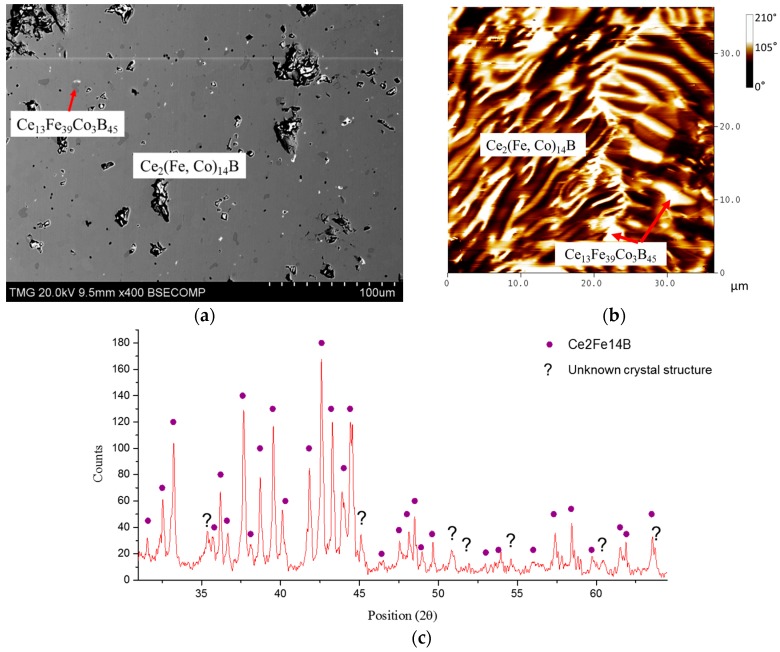
(**a**) BSE image of KA 1; (**b**) MFM image of KA 1; (**c**) XRD spectrum of KA 1.

Two phases formed in KA 1 (Ce_14_Fe_73_Co_7_B_6_) after annealing at 900 °C for 25 days, as shown in Figure 12a. The solid solubility of the dominating Ce_2_(Fe, Co)_14_B was measured as 6 at. % Co. A boron-rich compound (Ce_13_Fe_39_Co_3_B_45_) with similar chemical composition, which is found in the diffusion-couple study, is also observed in this key alloy. From the MFM test, as presented in Figure 12b, dominating stripe magnetic domain patterns with some dispersed non-magnetic plates were observed. Comparing the phase morphology between SEM and MFM images, it is concluded that the dominating Ce_2_(Fe, Co)_14_B phase is magnetic, and non-magnetic regions belong to Ce_13_Fe_39_Co_3_B_45_. In Figure 12c, Ce_2_(Fe, Co)_14_B is positively identified in the XRD pattern of KA 1. However, the crystal structure prototype of Ce_13_Fe_39_Co_3_B_45_ has not been reported, hence this compound cannot be verified by XRD analysis. A number of unlabeled peaks, marked by “?” in Figure 12c, may belong to Ce_13_Fe_39_Co_3_B_45_.

**Figure 13:**
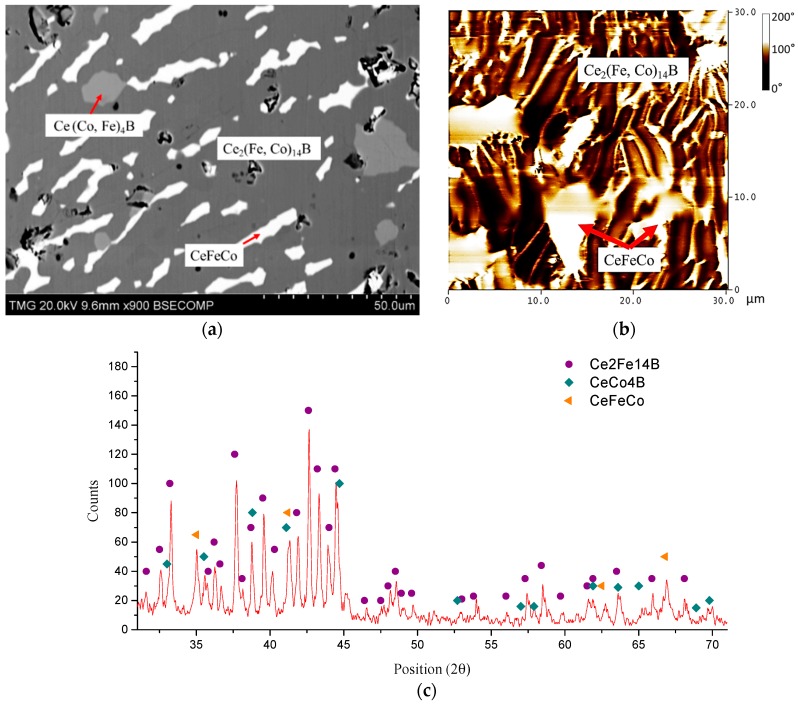
(**a**) BSE image of KA 2; (**b**) MFM image of KA 2; (**c**) XRD spectrum of KA 2.

When the concentration of Co in the global composition increased from 7 at. % in KA 1–12 at. % in KA 2, three-phase equilibrium between Ce_2_(Fe, Co)_14_B, Ce(Co, Fe)_4_B, and CeFeCo occurred, as shown in Figure 13a. Unlike in KA 1, the boron-rich phase is not observed in KA 2. Instead, a limited quantity of Ce(Co, Fe)_4_B started to form when the Co content was increased. XRD analysis also confirmed the phase constituents, as shown in Figure 13c. The solid solubility limit of Ce(Co, Fe)_4_B was measured as 54 at. % Fe, which can be considered as the maximum solid solubility of this phase in the Ce-Fe-Co-B system at 900 °C. In the MFM image, as shown in Figure 13b, it is clear that the magnetic domain pattern corresponds to the dominating Ce_2_(Fe, Co)_14_B. However, Ce(Co, Fe)_4_B cannot be easily distinguished from this image, which could be due to its relatively small amount. Another possibility is that Ce(Co, Fe)_4_B and Ce_2_(Fe, Co)_14_B have a similar magnetic domain pattern, which makes it difficult to distinguish them from each other. By comparing the morphology of CeFeCo in Figure 13a with non-magnetic islands in Figure 13b, this non-magnetic phase belongs to the CeFeCo compound.

**Figure 14:**
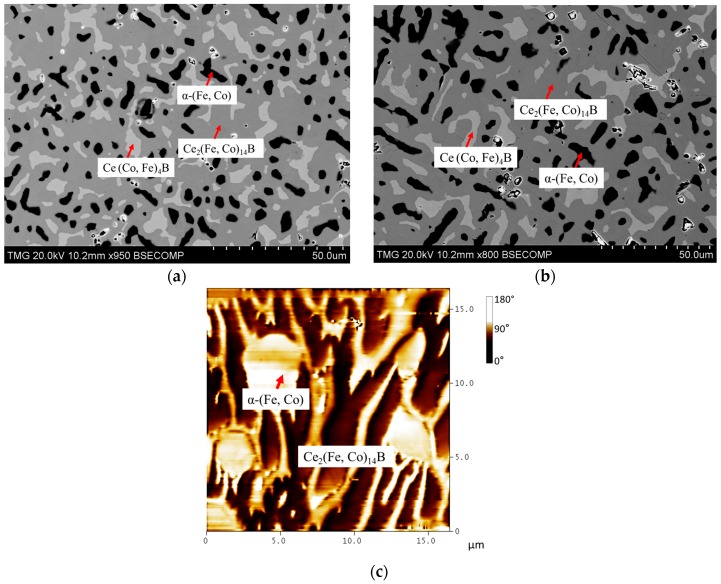
(**a**) BSE image of KA 4; (**b**) BSE image of KA 5; (**c**) MFM image of KA 5.

**Figure 15:**
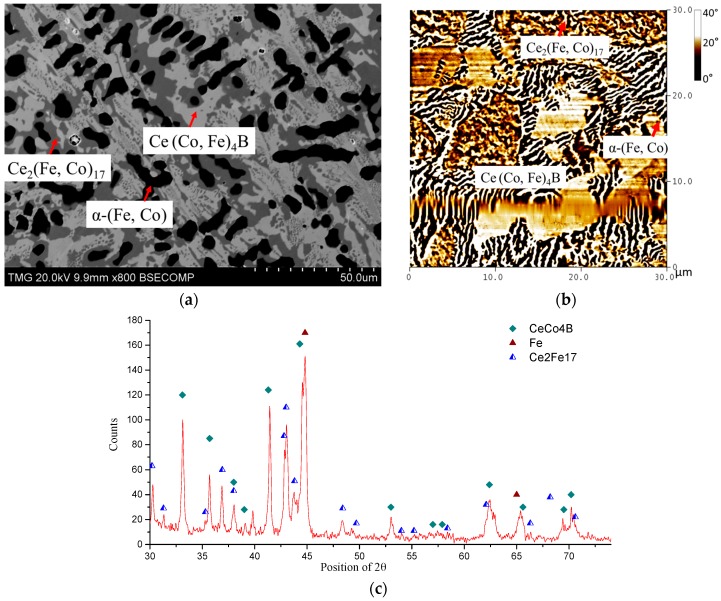
(**a**) BSE image of KA 6; (**b**) MFM image of KA 6; (**c**) XRD spectrum of KA 6.

**Figure 16:**
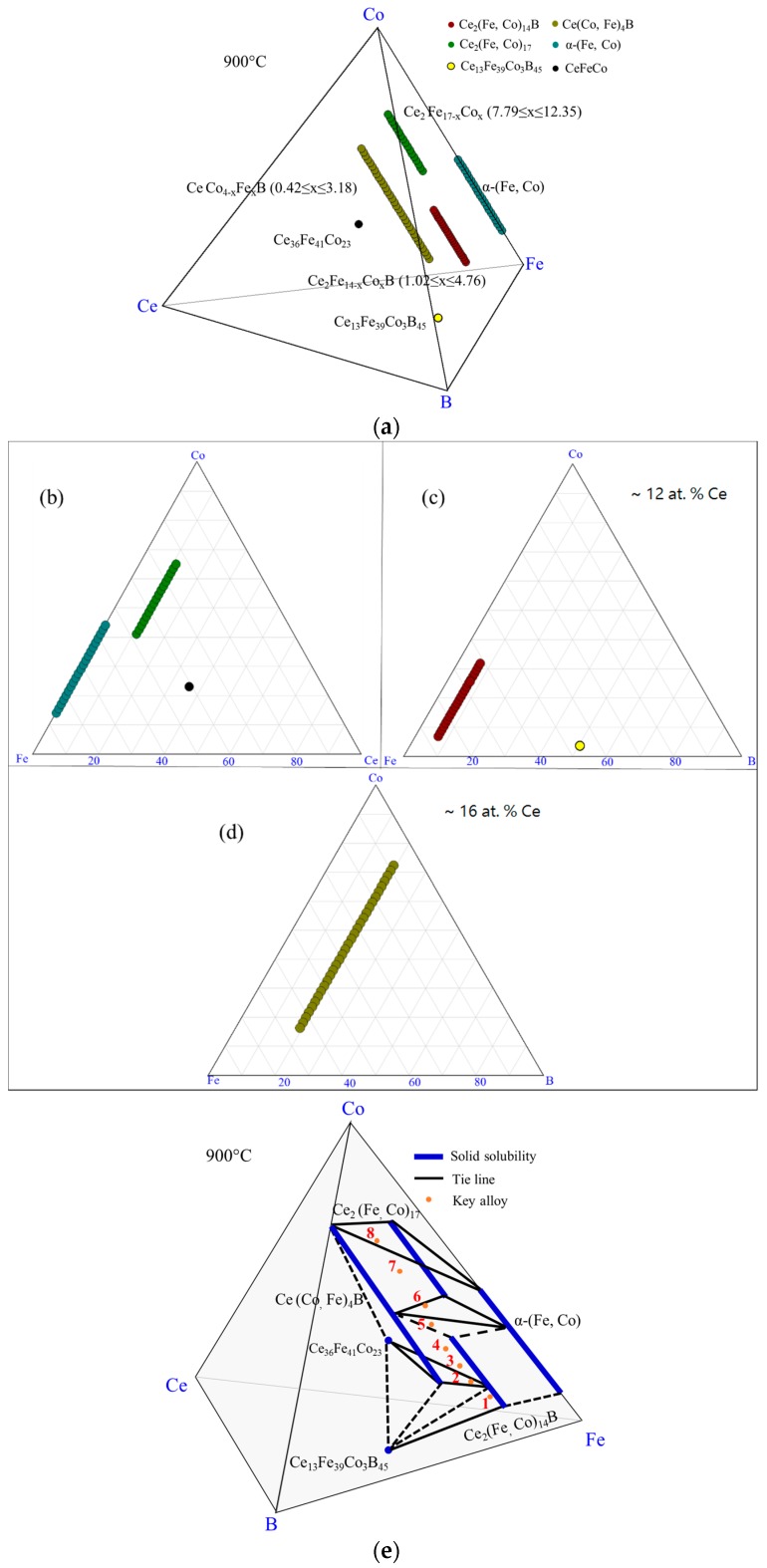
Homogeneity ranges of Ce_2_(Fe, Co)_14_B, and Ce(Co, Fe)_4_B obtained from key alloys: (**a**) 3D presentation of the experimental results; (**b**) Fe-Ce-Co ternary system; (**c**) pseudo ternary section at ~12 at. % Ce; (**d**) pseudo ternary section at ~16 at. % Ce; (**e**) phase relations obtained from KAs 1–8.

Ce_2_(Fe, Co)_14_B formed through peritectic solidification. Due to the sluggish kinetics of the peritectic reaction, the transformation of α-(Fe, Co) and CeFeCo to Ce_2_(Fe, Co)_14_B and Ce(Co, Fe)_4_B takes a very long time. Therefore, two-phase equilibrium was established between Ce_2_(Fe, Co)_14_B and Ce(Co, Fe)_4_B in KAs 3, 4, and 5. In KA 3, Co dissolved in Ce_2_Fe_14_B was measured as 18 at. %. With global Co concentrations increased in KAs 4 and 5, the solid solubility of Co in Ce_2_Fe_14_B was also further extended. Dominating Ce_2_(Fe, Co)_14_B was found in KA 4 (Ce_15_Fe_54_Co_24_B_7_), as shown in Figure 14a. Solid solubility of Ce_2_(Fe, Co)_14_B was measured as 22 at. % Co, which is consistent with the results obtained from DC2. However, with an increase in Co content to 32 at. % in KA 5 (Ce_14_Fe_46_Co_32_B_8_), Ce_2_(Fe, Co)_14_B was still found to form in this alloy, and solid solubility was measured as 28 at. % Co. The BSE image of KA 5 is presented in Figure 14b. Magnetic domains of Ce_2_(Fe, Co)_14_B can still be observed in both samples based on the MFM study, as can be seen in Figure 14c. KAs 6, 7, and 8 were prepared in the Co-rich region. The compositions of the detected phases are listed in Table 2. When Co content in KA 6 (Ce_12_Fe_42_Co_40_B_6_) reached 40 at. %, the Ce_2_(Fe, Co)_14_B phase disappeared. Whereas, Ce(Co, Fe)_4_B became the dominating phase and Ce_2_(Co, Fe)_17_ started to form in KA 6. KAs 7 and 8 confirmed the phase equilibria obtained from KA 6. In the XRD spectrum of KA 6 in Figure 15c, all three phases were confirmed by XRD. Three-phase regions Ce(Co, Fe)_4_B, α-(Fe, Co), and Ce_2_(Co, Fe)_17_ were established from these samples, as presented in Figure 16e. Moreover, the dominating Ce(Co, Fe)_4_B was found to be magnetic, as shown in Figure 15b. Compared to the MFM images of the Ce_2_(Fe, Co)_14_B phase in KAs 1–5, the magnetic contrast of Ce(Co, Fe)_4_B is significantly lower. The magnetic contrast of Ce(Co, Fe)_4_B in Figure 15b is in the range of 0–40 degree; whereas, the magnetic contrast of Ce_2_(Fe, Co)_14_B in KAs 1–5 is in the range of 0–180 degrees or higher. This indicates that the magnetization of Ce(Co, Fe)_4_B is weaker than that of Ce_2_(Fe, Co)_14_B. In KA 6, there is a number of weaker closure domain patterns at the top and middle of Figure 15b, which belong to Ce_2_(Co, Fe)_17_. The non-magnetic regions were considered as α-(Fe, Co) which were consistent with the MFM studies of other key alloys. The MFM results are only considered as indications of the effect of composition on the magnetic domains. A detailed study of the intrinsic magnetic properties of these phases will soon be published elsewhere.

From the key alloys study, the homogeneity ranges of Ce_2_(Fe, Co)_14_B and Ce(Co, Fe)_4_B were measured as 28 at. % Co and 54 at. % Fe, respectively. They were presented by Ce_2_Fe_14−*x*_Co*_x_*B (1.02 ≤ *x* ≤ 4.76) and CeCo_4−*x*_Fe*_x_*B (0.42 ≤ *x* ≤3.18) formulae. The experimental results of eight key alloys are summarized in Figure 16a. Solid solubility of ε_1_ (Ce_2_(Fe, Co)_17_) observed in the diffusion couples was confirmed by key alloys 6, 7, and 8. The Co dissolved in ε_1_ was found to extend further, up to 65 at. % in Ce_2_(Fe, Co)_17_, as presented by the Ce_2_Fe_17−*x*_Co*_x_* (7.79 ≤ *x* ≤ 12.35) formula and shown in Figure 16. In the current study, it is found that Ce(Co, Fe)_4_B only formed in an Fe-rich region when Co content is greater than 10 at. %. When Co content is below 10 at. %, the boron-rich phase, Ce_13_Fe_39_Co_3_B_45_, started to form. Moreover, Ce(Co, Fe)_4_B might be a weaker magnetic phase compared to Ce_2_(Fe, Co)_14_B, based on the MFM results. The phases formed in the Fe-Co-Ce system are presented in Figure 16b. Two pseudo ternary sections at 12 at. % and 16 at. % Ce were used to demonstrate the locations of Ce_2_(Fe, Co)_14_B, and Ce(Co, Fe)_4_B, respectively, which can be seen in Figure 16c,d. The phase equilibria obtained from eight key alloys are presented in Figure 16e. The dotted lines indicate the possible phase equilibria between the detected phases. Two three-phase equilibria (Ce_2_(Fe, Co)_14_B/Ce(Co, Fe)_4_B/CeFeCo and Ce(Co, Fe)_4_B/α-(Fe, Co)/Ce_2_(Fe, Co)_17_) were established.

**Figure 17:**
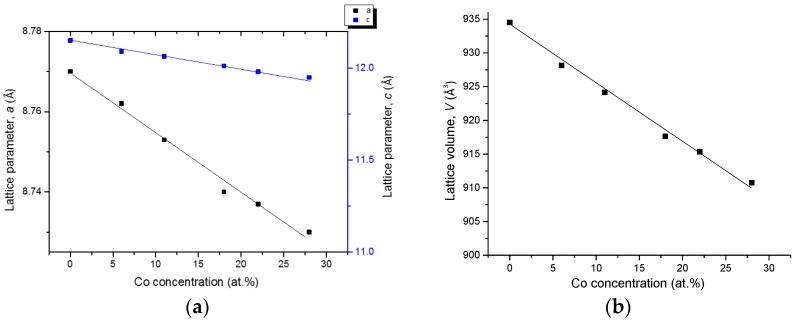
Cell parameters *a* and *c* (**a**) and lattice volume *V* (**b**) with Co concentration for the Ce_2_(Fe, Co)_14_B from KAs 1–5.

Ce_2_(Fe, Co)_14_B forms a substitutional solid solution in this quaternary system, where Co substitutes for Fe atoms, while Ce and B contents remain constant as 12 at. % and 6 at. %, respectively. The maximum solid solubility of Ce_2_(Fe, Co)_14_B has been determined by SEM/WDS as 28 at. % Co in KA 5 at 900 °C. All XRD data has shown that this solid solution, in KAs 1–5, crystallized in a tetragonal structure with a *P42/mnm* (68) space group and an Nd_2_Fe_14_B prototype. Figure 17 shows the cell parameters variations with Co concentration in the selected key alloys. The peak positions shift to a higher angle with increasing Co content. The substitution of Fe with Co, which has a smaller atomic radius, decreases the unit cell parameters and lattice volumes. This is confirmed by the increase in 2θ values of the peak positions from KAs 1–5 due to the increasing Co concentration. The linear relation between the lattice parameters, lattice volume, and Co concentration obey Vegard’s law [30], indicating clearly the occurrence of substitution solid solubility in the Ce_2_(Fe, Co)_14_B, as plotted in Figure 17.

**Figure 18:**
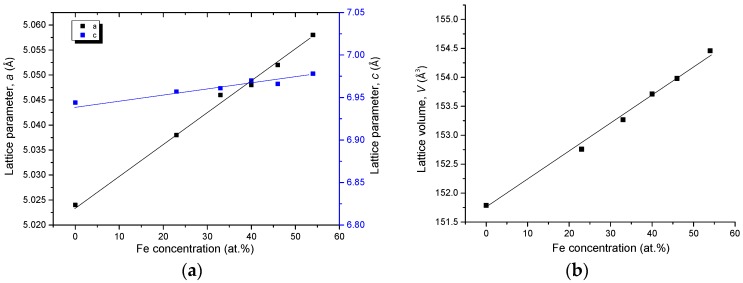
Cell parameters *a* and *c* (**a**) and lattice volume *V* (**b**) with Co concentration for the Ce(Co, Fe)_4_B from KAs 2–6.

The unit cell parameters of Ce(Co, Fe)_4_B from KAs 2–6 are plotted in Figure 18. Similar to Ce_2_(Fe, Co)_14_B, CeCo_4_B also forms a substitutional solid solution in this quaternary system, while Ce and B contents remain constant as 16 at. % and 17 at. %, respectively. The substitution of Co with Fe, which has a larger atomic radius, increases the unit cell parameters. Cell lengths *a* and *c* reach maximum when CeCo_4_B has the maximum quaternary solid solution of 54 at. % Fe in KA 2 at 900 °C. When Fe content in CeCo_4_B is reduced, the cell parameters are also decreased. This is confirmed by the increase in the 2θ values of the peaks positions from the KAs 2–8 due to the decreases in the Fe concentration. The XRD results have demonstrated that Ce(Co, Fe)_4_B crystallized in a hexagonal structure with a *P6/mnm* (191) space group and a CeCo_4_B prototype. The linear relation between the lattice parameters, lattice volume, and Co concentration obey Vegard’s law [30], indicating again the occurrence of substitution solid solubility in the Ce(Co, Fe)_4_B, as plotted in Figure 18.

#### 4.3.2. Homogeneity ranges of Ce(Co, Fe)_12_B_6_ and Ce_3_(Co, Fe)_11_B_4_

Solid solubilities of Ce(Co, Fe)_12_B_6_ and Ce_3_(Co, Fe)_11_B_4_ were measured as 18 at. % Fe and 23 at. % Fe at 900 °C in the diffusion couple analysis. Seven key alloys were designed to verify the results obtained from the diffusion couples and determine the solubility limits of these two phases in the Ce-Fe-Co-B system at 900 °C. The chemical compositions of the key alloys prepared for this purpose are listed in Table 3. Phase equilibria determined from these key alloys are also summarized in Table 3. The actual global compositions of the samples were determined by EDS area mapping. Three maps were taken for each sample, and the differences in three scans were less than 2 at. % for all the elements.

**Figure 19:**
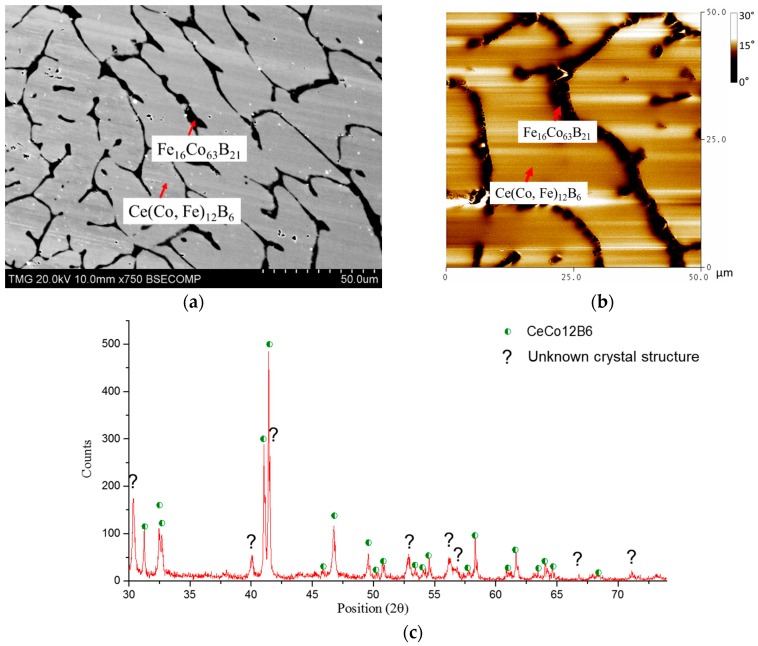
(**a**) BSE image of KA 9; (**b**) MFM image of KA 9; (**c**) XRD spectrum of KA 9.

Ce(Co, Fe)_12_B_6_ was first found in the diffusion couple study and the homogeneity range was measured as 9–18 at. % Fe. KA 9 was prepared in the Co-rich side with Ce_6_Fe_6_Co_58_B_30_ chemical composition. After annealing at 900 °C for 25 days, the dominating Ce(Co, Fe)_12_B_6_ was obtained and some precipitates with Fe_16_Co_63_B_21_ chemical composition were also observed, as shown in Figure 19a. Fe dissolved in CeCo_12_B_6_ was determined as 4 at. %. From the MFM image in Figure 19b, the dominating phase was found to be non-magnetic, which is also consistent with the MFM result of DC4. The magnetic domain found in this image belongs to Fe_16_Co_63_B_21_. Based on the XRD analysis shown in Figure 19c, the dominating Ce(Co, Fe)_12_B_6_ phase was positively identified. However, the crystal structure prototype of Fe_16_Co_63_B_21_ is not available in the literature. Therefore, this phase cannot be verified by XRD analysis. Moreover, there are some unlabeled peaks, marked with “?” in Figure 19c, which may belong to Fe_16_Co_63_B_21_.

**Figure 20:**
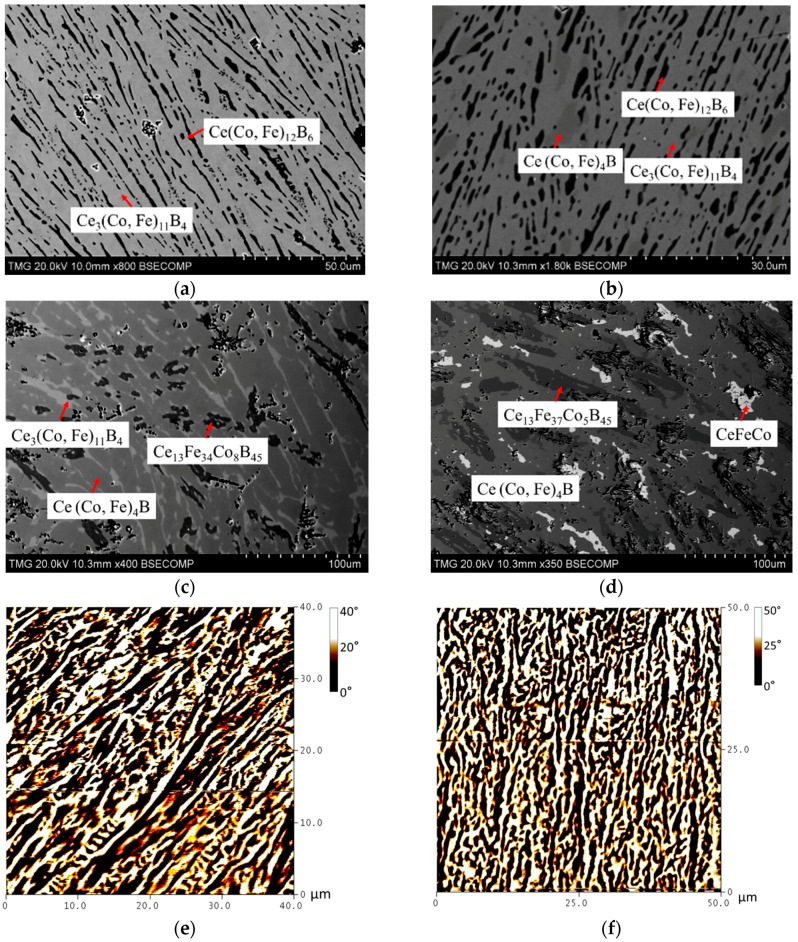

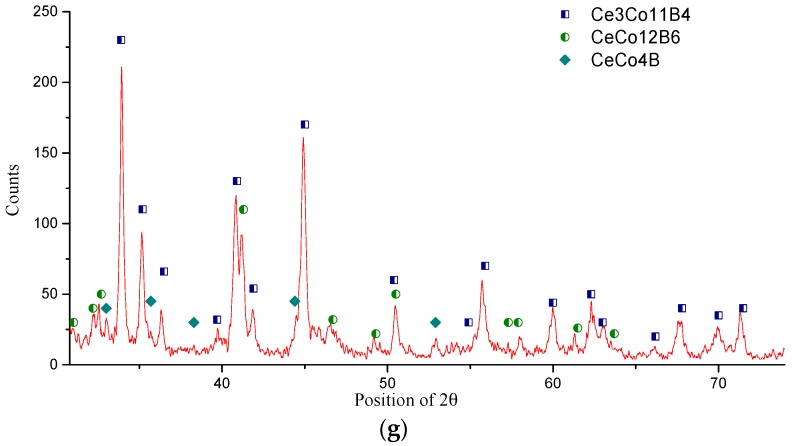
(**a**) BSE image of KA 11; (**b**) BSE image of KA 12; (**c**) BSE image of KA 13; (**d**) BSE image of KA 14; (**e**) MFM image of KA 11; (**f**) MFM image of KA 12; (**g**) XRD spectrum of KA 12.

KAs 10–14 were selected to measure the maximum solid solubility of Ce(Co, Fe)_12_B_6_ and Ce_3_(Co, Fe)_11_B_4_. These five key alloys were prepared along the homogeneity range of Ce_3_(Co, Fe)_11_B_4_ by substituting a different amount of Fe with Co, which were presented by the formulae Ce_3_Co_11 −_ *_x_*Fe*_x_*B_4_ with *x* = 1.44, 3.24, 5.94, 7.38, and 9.00. The phase relations between Ce_3_(Co, Fe)_11_B_4_, Ce(Co, Fe)_12_B_6_, and Ce(Co, Fe)_4_B were investigated. As shown in Figure 20a, two-phase equilibrium between Ce_3_(Co, Fe)_11_B_4_ and Ce(Co, Fe)_12_B_6_ was established from KA 11. The dominating Ce_3_(Co, Fe)_11_B_4_ phase was identified and the black precipitates in Figure 20a were found to be Ce(Co, Fe)_12_B_6_. When the global Fe concentration in KA 12 was increased to 33 at. %, a small amount of the Ce(Co, Fe)_4_B phase appeared, as can be seen in Figure 20b. Fe dissolved in Ce_3_(Co, Fe)_11_B_4_ and Ce(Co, Fe)_12_B_6_ was measured as 28 at. % and 46 at. %, respectively. The detected phases were all confirmed by XRD, as presented in Figure 20g. As Fe content reached 42 at. % in KA 13, Ce(Co, Fe)_12_B_6_ disappeared and Ce(Co, Fe)_4_B became the dominating phase, along with a limited amount of Ce_3_(Co, Fe)_11_B_4_.

**Figure 21:**
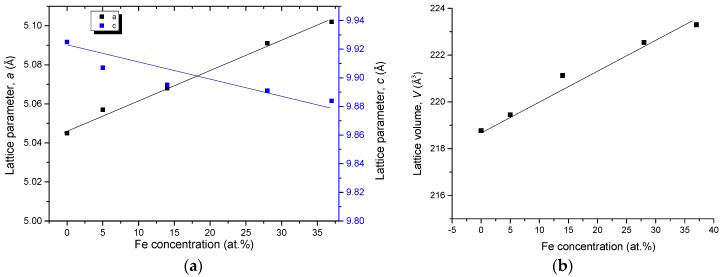
Cell parameters *a* and *c* (**a**) and lattice volume *V* (**b**) with Co concentration for the Ce_3_(Co, Fe)_11_B_4_ from KAs 10–13.

When Fe content was increased to 50 at. % in KA 14, Ce_3_(Co, Fe)_11_B_4_ completely disappeared and three-phase equilibrium Ce(Co, Fe)_4_B, Ce_13_Fe_37_Co_5_B_45_, and CeFeCo was established. Maximum solid solubility of Ce_3_(Co, Fe)_11_B_4_ was measured as 37 at. % Fe in KA 13. From the MFM results, as shown in Figure 20e,f, a mixture of stripe and closure domain patterns is observed, indicating that the dominating Ce_3_(Co, Fe)_11_B_4_ dissolving 14 at. % Fe and 28 at. % Fe, respectively, is a magnetic phase. Magnetic Ce_3_(Co, Fe)_11_B_4_ was first found in DC4, and is now confirmed by the MFM study of the key alloys. The substitution of Co with Fe in Ce_3_(Co, Fe)_11_B_4_ is also studied by XRD. Compared to Co, Fe has a larger atomic radius, which could increase the unit cell parameters. As can be seen in Figure 21, cell length *a* and lattice volume *V* reach maximum when Ce_3_(Co, Fe)_11_B_4_ has the maximum quaternary solid solubility of 37 at. % Fe in KA 13 at 900 °C. However, when Fe concentration in Ce_3_(Co, Fe)_11_B_4_ was increased, the cell parameter *c* decreased to some extent. Nevertheless, the volume of the unit cell increased with Fe content, indicating the overall effect of larger atom substitution. The XRD results have demonstrated that Ce_3_(Co, Fe)_11_B_4_ crystallizes in a hexagonal structure with a *P6/mnm* (191) space group and a Ce_3_Co_11_B_4_ prototype. The linear relation between the lattice parameters, lattice volume, and Co concentration indicates the occurrence of substitution solid solubility, according to the Vegard’s law [30], as shown in Figure 21.

In KAs 13 and 14, a boron-rich phase with Ce_13_Fe_34_Co_8_B_45_ and Ce_13_Fe_37_Co_5_B_45_ chemical compositions was identified. This phase was first observed in DCs 3 and 4, with Ce_13_Fe_38_Co_4_B_45_ and Ce_13_Fe_32_Co_10_B_45_, respectively. Similar results were also obtained from KA 1, but with Ce_13_Fe_39_Co_3_B_45_ composition. Analyzing these compositions reveals that Ce and B contents are consistent and the compositional difference was due to Fe/Co atomic exchange. Moreover, the unidentified peaks in the XRD spectra of KAs 1, 13, and 14 were repeatable, but no reported crystal structure prototype could be found for this phase. Thus, we consider this boron-rich phase, Ce_13_Fe*_x_*Co*_y_*B_45_ (32 ≤ *x* ≤ 39, 3 ≤ *y* ≤ 10), as a new quaternary solid solution in the Ce-Fe-Co-B system at 900 °C.

**Figure 22:**
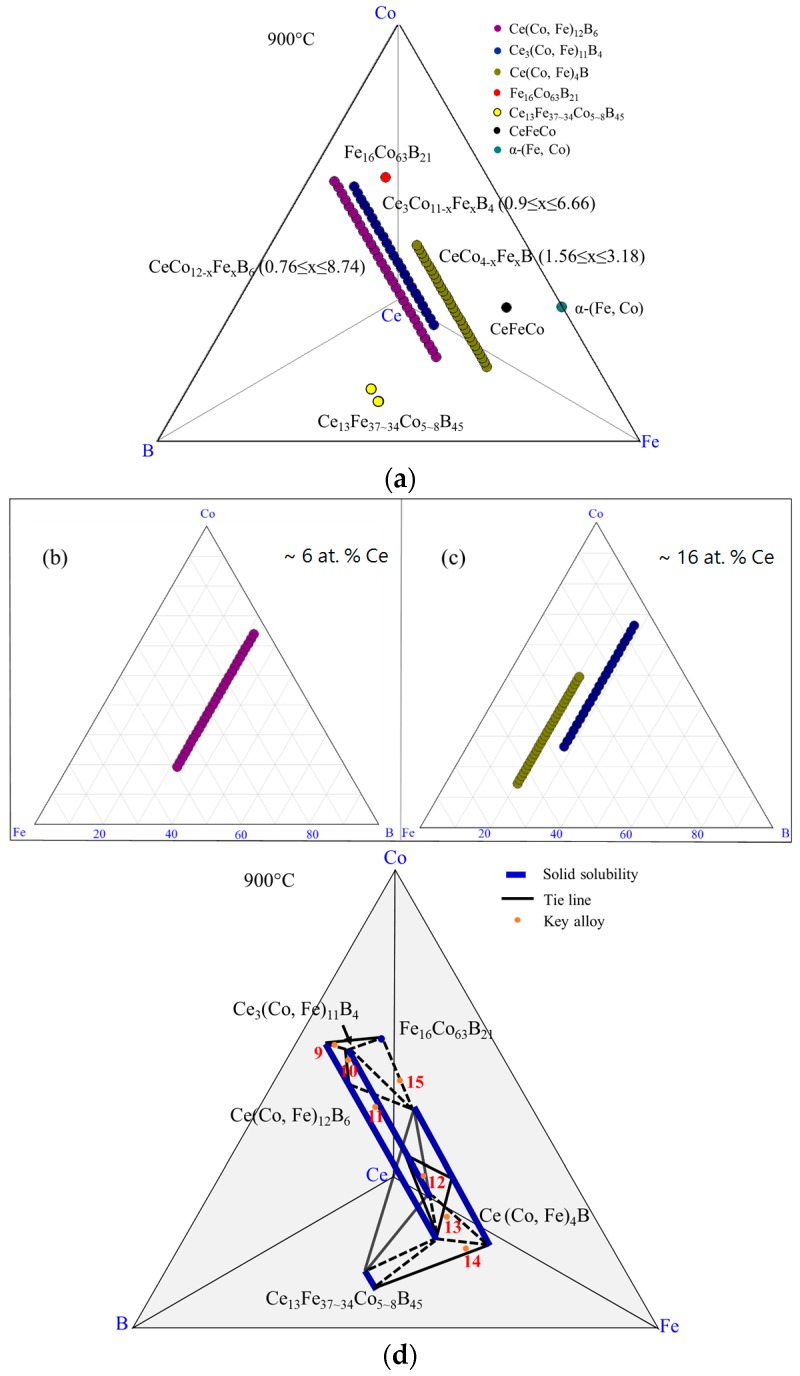
Homogeneity ranges of Ce_3_(Co, Fe)_11_B_4_ and Ce(Co, Fe)_12_B_6_ obtained from key alloys: (**a**) 3D presentation of the experimental results; (**b**) pseudo ternary section at ~6 at. % Ce; (**c**) pseudo ternary section at ~16 at. % Ce; (**d**) phase relations obtained from the key alloys.

The experimental results of KAs 9–15 are summarized in Figure 22a. Solid solubilities of magnetic Ce_3_(Co, Fe)_11_B_4_ and non-magnetic Ce(Co, Fe)_12_B_6_ were first measured by diffusion couples as 23 at. % Fe and 18 at. % Fe, respectively. During the key alloy study, it has been proven that these two phases further extended into this system. And the solubility limits were measured as 46 at. % Fe and 37 at. % Fe, respectively. They can be presented as CeCo_12−*x*_Fe*_x_*B_6_ (0.76 ≤ *x* ≤ 8.74) and Ce_3_Co_11−*x*_Fe*_x_*B_4_ (0.9 ≤ *x* ≤ 5.04), as illustrated in Figure 22a. Two pseudo ternary sections at 6 at. % and 16 at. % Ce were used to demonstrate the locations of Ce(Co, Fe)_12_B_6_ and Ce_3_(Co, Fe)_11_B_4_, respectively, in Figure 22b,c. In Figure 22d, solid lines are the phase equilibria determined from key alloys, and dotted lines indicate the possible phase equilibria of detected phases.

**Figure 23:**
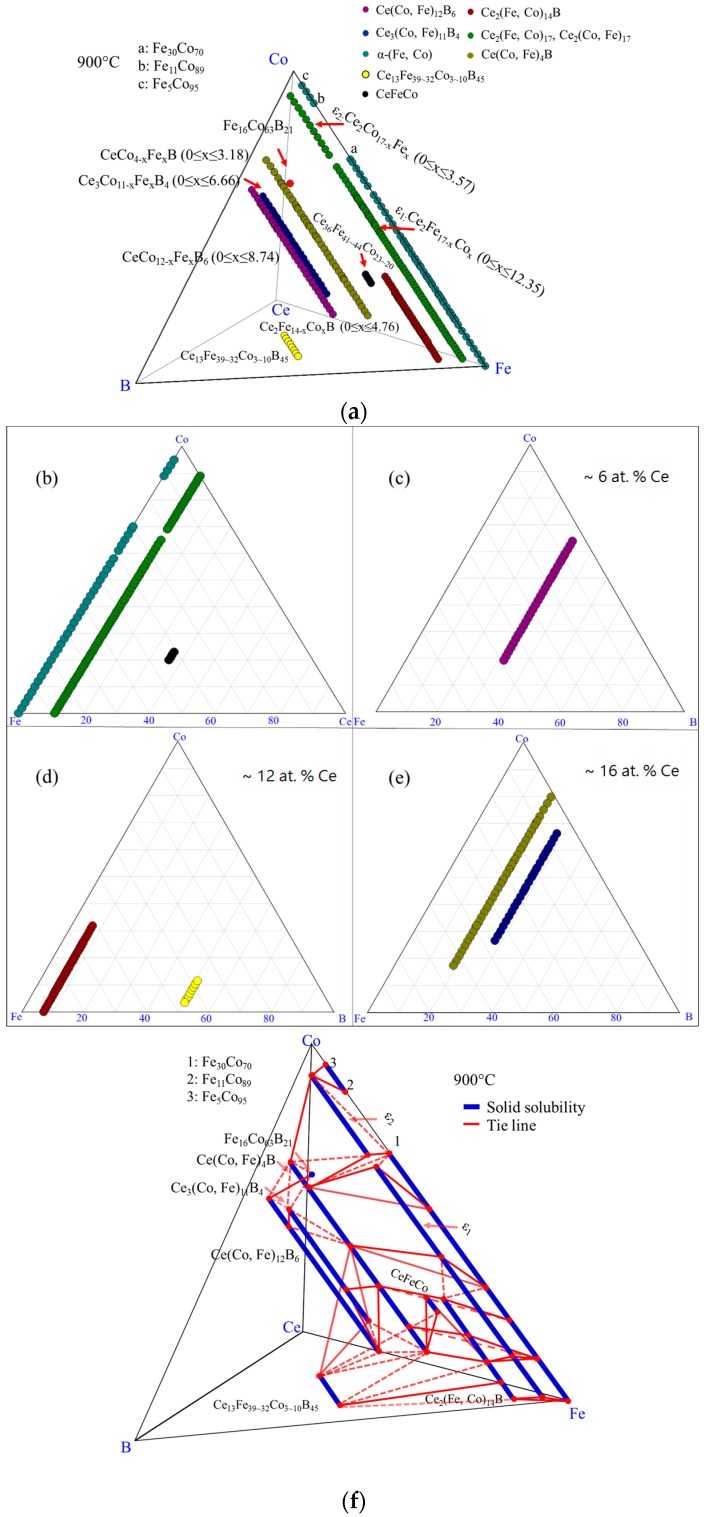
Combined experimental results of diffusion couples and key alloys in the Ce-Fe-Co-B system at 900 °C: (**a**) 3D presentation of the experimental results; (**b**) Fe-Ce-Co ternary system; (**c**) pseudo ternary section at ~6 at. % Ce; (**d**) pseudo ternary section at ~12 at. % Ce; (**e**) pseudo ternary section at ~16 at. % Ce; (**f**) phase relations in the Fe-rich region of the Ce-Fe-Co-B system at 900 °C.

Combining the results which were obtained from the diffusion couples with those attained from the key alloys, the phases which were detected in the Ce-Fe-Co-B system in the Fe-Co rich region at 900 °C were plotted in Figure 23a. Seven three-phase equilibria were established as: α-(Fe, Co)/Ce_2_(Fe, Co)_14_B/Ce_2_(Fe, Co)_17_; Ce_2_(Fe, Co)_14_B/Ce(Co, Fe)_4_B/CeFeCo; Ce_2_(Fe, Co)_17_/Ce(Co, Fe)_4_B/α-(Fe, Co); Ce_3_(Co, Fe)_11_B_4_/Ce(Co, Fe)_12_B_6_/Ce(Co, Fe)_4_B; Ce_3_(Co, Fe)_11_B_4_/Ce(Co, Fe)_12_B_6_/α-(Fe, Co); Ce_3_(Co, Fe)_11_B_4_/Ce(Co, Fe)_4_B/Ce_13_Fe_34_Co_8_B_45_, and Ce_3_(Co, Fe)_11_B_4_/Ce(Co, Fe)_4_B/α-(Fe, Co). The phase relations of the Ce-Fe-Co-B system in the Fe-rich corner at 900 °C were plotted, as shown in Figure 23f. Solid lines are the tie-lines determined in this study and dotted lines represent the possible phase relations. Among those detected phases, Ce_2_(Fe, Co)_14_B, Ce(Co, Fe)_4_B, and Ce_3_(Co, Fe)_11_B_4_ were magnetic phases in this system, which was proven by the MFM study. Also, Ce(Co, Fe)_12_B_6_ was identified as non-magnetic. The phases formed in the Fe-Co-Ce system are plotted in the triangle, as shown in Figure 23a. Three pseudo ternary sections at around 6 at. %, 12 at. % and 16 at. % Ce were constructed to demonstrate the locations and phase relations of Ce(Co, Fe)_12_B_6_, Ce_2_(Fe, Co)_14_B, Ce(Co, Fe)_4_B, and Ce_3_(Co, Fe)_11_B_4_.

## 5. Conclusions

A high-throughput screening method was found to be effective in studying the phase equilibria in the Ce-Fe-Co-B system while exploring potential magnetic phases. Phase equilibria and homogeneity ranges have been determined in the Fe-Co rich side of the Ce-Fe-Co-B system at 900 °C. Three magnetic phases are observed which are presented as Ce_2_Fe_14−*x*_Co*_x_*B (0 ≤ *x* ≤ 4.76), CeCo_4−*x*_Fe*_x_*B (0 ≤ *x* ≤ 3.18), and Ce_3_Co_11−*x*_Fe*_x_*B_4_ (0 ≤ *x* ≤ 6.66). Ce_2_(Fe, Co)_14_B exhibited stronger magnetic interaction than Ce(Co, Fe)_4_B and Ce_3_(Co, Fe)_11_B_4_ during the MFM analysis. Moreover, a non-magnetic CeCo_12−*x*_Fe*_x_*B_6_ (0 ≤ *x* ≤ 8.74) was found to form in this system at 900 °C. A boron-rich solid solution with Ce_13_Fe*_x_*Co*_y_*B_45_ (32 ≤ *x* ≤ 39, 3 ≤ *y* ≤ 10) chemical composition was observed in this quaternary system. The crystal structure of this phase has not been reported in the literature. In addition, two solid solutions ε_1_ (Ce_2_Fe_17−*x*_Co*_x_* (0 ≤ *x* ≤ 12.35)) and ε_2_ (Ce_2_Co_17−*x*_Fe*_x_* (0 ≤ *x* ≤ 3.57)) were found to form between Ce_2_Fe_17_ and Ce_2_Co_17_ in the Ce-Fe-Co ternary system at 900 °C.

## Figures and Tables

**Table 1:** Chemical compositions across the diffusion couples and the corresponding phases.

Diffusion Couple	Layer	Composition (at. %)				Corresponding Phase
		Ce	Fe	Co	B	
DC1 (Ce_2_Fe_14_B/Co)	1	12	82–73	0–9	6	Ce_2_(Fe, Co)_14_B
		0	100–93	0–7	0	α-(Fe, Co)
	2	11	79–34	10–55	0	Ce_2_(Fe, Co)_17_
		0	93–57	7–43	0	α-(Fe, Co)
	3	16	9	59	16	Ce(Co, Fe)_4_B
		0	57–39	43–61	0	α-(Fe, Co)
	4	11	19	70	0	Ce_2_(Co, Fe)_17_
		0	39	61	0	α-(Fe, Co)
	5	0	35–30	65–70	0	α-(Fe, Co)
	6	0	12	88	0	γ-(Fe, Co)
		11	2	87	0	Ce_2_(Co, Fe)_17_
DC2 (Ce_13_Fe_80_B_7_/Co_90_Ce_10_)	1	12	82–60	0–22	6	Ce_2_(Fe, Co)_14_B
		0	100–82	0–18	0	α-(Fe, Co)
	2	15	27–17	42–52	16	Ce(Co, Fe)_4_B
		0	82–37	18–63	0	α-(Fe, Co)
	3	0	35–33	65–67	0	α-(Fe, Co)
	4	16	6–1	63–68	15	Ce(Co, Fe)_4_B
		11	14	75	0	Ce_2_(Co, Fe)_17_
	5	0	11–0	89–100	0	γ-(Fe, Co)
		11	14–0	75–89	0	Ce_2_(Co, Fe)_17_
DC3 (Ce_10_Fe_75_B_15_/Co)	1	12	82–65	0–17	6	Ce_2_(Fe, Co)_14_B
		0	100–70	0–30	0	α-(Fe, Co)
		13	38	4	45	Ce_13_Fe_38_Co_4_B_45_
	2	16	21–5	40–56	23	Ce_3_(Co, Fe)_11_B_4_
		0	70–44	30–56	0	α-(Fe, Co)
	3	6	11	53	30	Ce(Co, Fe)_12_B_5_
	4	0	33–30	67–70	0	α-(Fe, Co)
DC4 (Ce_15_Fe_43_Co_19_B_23_/Co)	1	16	45–40	23–28	16	Ce(Co, Fe)_4_B
		0	17	83	0	α-(Fe, Co)
		13	32	10	45	Ce_13_Fe_32_Co_10_B_45_
	2	17	23–9	37–51	23	Ce_3_(Co, Fe)_11_B_4_
		0	65–53	35–47	0	α-(Fe, Co)
	3	6	18–9	46–55	30	Ce(Co, Fe)_12_B_5_
		0	53	47	0	α-(Fe, Co)
	4	0	31	69	0	α-(Fe, Co)
	5	0	14–11	86–89	0	γ-(Fe, Co)

**Table 2:** Chemical composition of key alloys and detected phases.

Key Alloys Number	Actual Global Composition (at. %)				WDS Composition (at. %)				Corresponding Phases	
	Ce	Fe	Co	B	Ce	Fe	Co	B	By WDS	By XRD
KA 1	14	73	7	6	12	76	6	6	Ce_2_(Fe, Co)_14_B	Ce_2_(Fe, Co)_14_B
					13	39	3	45	Ce_13_Fe_39_Co_3_B_45_	N/A *
KA 2	15	66	12	7	12	71	11	6	Ce_2_(Fe, Co)_14_B	Ce_2_(Fe, Co)_14_B
					16	54	15	15	Ce(Co, Fe)_4_B	Ce(Co, Fe)_4_B
					36	41	23	0	CeFeCo	CeFeCo
KA 3	14	58	20	8	12	64	18	6	Ce_2_(Fe, Co)_14_B	Ce_2_(Fe, Co)_14_B
					16	46	23	15	Ce(Co, Fe)_4_B	Ce(Co, Fe)_4_B
					0	86	14	0	α-(Fe, Co)	α-(Fe, Co)
KA 4	15	54	24	7	12	60	22	6	Ce_2_(Fe, Co)_14_B	Ce_2_(Fe, Co)_14_B
					16	40	28	16	Ce(Co, Fe)_4_B	Ce(Co, Fe)_4_B
					0	80	20	0	α-(Fe, Co)	α-(Fe, Co)
KA 5	14	46	32	8	12	54	28	6	Ce_2_(Fe, Co)_14_B	Ce_2_(Fe, Co)_14_B
					16	33	35	16	Ce(Co, Fe)_4_B	Ce(Co, Fe)_4_B
					0	76	24	0	α-(Fe, Co)	α-(Fe, Co)
KA 6	12	42	40	6	11	48	41	0	Ce_2_(Fe, Co)_17_	Ce_2_(Fe, Co)_17_
					16	23	45	16	Ce(Co, Fe)_4_B	Ce(Co, Fe)_4_B
					0	66	34	0	α-(Fe, Co)	α-(Fe, Co)
KA 7	12	32	50	6	11	37	52	0	Ce_2_(Fe, Co)_17_	Ce_2_(Fe, Co)_17_
					17	13	54	16	Ce(Co, Fe)_4_B	Ce(Co, Fe)_4_B
					0	57	43	0	α-(Fe, Co)	α-(Fe, Co)
KA 8	12	22	60	6	11	24	65	0	Ce_2_(Fe, Co)_17_	Ce_2_(Fe, Co)_17_
					17	7	60	16	Ce(Co, Fe)_4_B	Ce(Co, Fe)_4_B
					0	56	44	0	α-(Fe, Co)	α-(Fe, Co)

* Not available: Unknown crystal structure which could not be confirmed by XRD.

**Table 3:** Chemical composition of key alloys and detected phases.

Key Alloys Number	Actual Global Composition (at. %)				WDS Composition (at. %)				Corresponding Phases	
	Ce	Fe	Co	B	Ce	Fe	Co	B	By WDS	By XRD
KA 9	9	7	56	28	6	4	60	30	Ce(Co, Fe)_12_B_6_	Ce(Co, Fe)_12_B_6_
					0	16	63	21	Fe_16_Co_63_B_21_	N/A *
KA 10	16	8	51	25	17	5	55	23	Ce_3_(Co, Fe)_11_B_4_	Ce_3_(Co, Fe)_11_B_4_
					6	14	51	29	Ce(Co, Fe)_12_B_6_	Ce(Co, Fe)_12_B_6_
KA 11	17	18	43	22	17	14	46	23	Ce_3_(Co, Fe)_11_B_4_	Ce_3_(Co, Fe)_11_B_4_
					6	32	32	30	Ce(Co, Fe)_12_B_6_	Ce(Co, Fe)_12_B_6_
KA 12	17	33	27	23	17	28	32	23	Ce_3_(Co, Fe)_11_B_4_	Ce_3_(Co, Fe)_11_B_4_
					6	46	18	30	Ce(Co, Fe)_12_B_6_	Ce(Co, Fe)_12_B_6_
					17	39	27	17	Ce(Co, Fe)_4_B	Ce(Co, Fe)_4_B
KA 13	17	42	18	23	17	37	23	23	Ce_3_(Co, Fe)_11_B_4_	Ce_3_(Co, Fe)_11_B_4_
					16	47	20	17	Ce(Co, Fe)_4_B	Ce(Co, Fe)_4_B
					13	34	8	45	Ce_13_Fe_34_Co_8_B_45_	N/A *
KA 14	17	50	10	23	16	55	12	17	Ce(Co, Fe)_4_B	Ce(Co, Fe)_4_B
					13	37	5	45	Ce_13_Fe_37_Co_5_B_45_	N/A *
					35	45	20	0	CeFeCo	CeFeCo
KA 15	15	20	49	16	17	16	44	23	Ce_3_(Co, Fe)_11_B_4_	Ce_3_(Co, Fe)_11_B_4_
					16	26	42	16	Ce(Co, Fe)_4_B	Ce(Co, Fe)_4_B
					0	68	32	0	α-(Fe, Co)	α-(Fe, Co)

* Not available: Unknown crystal structure which could not be confirmed by XRD.
